# Acute Kidney Injury and Hair-Straightening Products

**DOI:** 10.1016/j.ekir.2024.06.010

**Published:** 2024-06-10

**Authors:** Aurélie Huber, Christine Deffert, Solange Moll, Sophie de Seigneux, Lena Berchtold

**Affiliations:** 1 Nephrology service, University Hospitals of Geneva, Geneva, Switzerland; 2 Diagnostic service, University Hospitals of Geneva, Geneva, Switzerland; 3 Pathology service, University Hospitals of Geneva, Geneva, Switzerland

## Introduction

Acute kidney injury (AKI) is common and can affect 8.3% of ambulatory patients and up to 32% of hospitalized patients.^1^ The main causes of AKI are acute tubular necrosis, and prerenal and acute on chronic renal failure. Recently, Robert *et al.*^2^ describe a case of AKI presumed due to hair-straightening products containing glyoxylic acid. A mouse model revealed the presence of elongated calcium oxalate monohydrate crystals in urine, similar to those observed after ethylene glycol intoxication. These results provide evidence that hair-straightening creams containing glyoxylic acid may be responsible for calcium oxalate–induced nephropathy after hair-straightening procedures (Table 1). Only 27 such cases were previously published.^3^ Here, we report a clinical case in Switzerland.

**Table 1:** Distinct teaching points^2^^,^^3^

Hair-straightening procedures are potential causes of oxalate nephropathy with acute kidney injury.
Symptoms may include nausea or vomiting, abdominal pain, and scalp irritation.
Early detection needs to be performed rapidly to manage and prevent chronic kidney disease.
There is a need for widespread awareness about kidney complications from these products.
Further studies are needed to study the epidemiology of acute kidney injury after the use of hair straightening products.

## Case Presentation

In September 2023, a 42-year-old Brazilian female patient presented at the University Hospital of Geneva, Switzerland, with symptoms of asthenia, nausea, vomiting, and right flank pain. She reported a 2-day history of diarrhea without fever. Her medical history was unremarkable. Physical examination revealed right-sided abdominal tenderness, with pain elicited upon palpation of the right kidney. She took acetaminophen and ibuprofen with partial relief of her symptoms. Vital signs included hypertension at 158/111 mm Hg and a heart rate of 87 beats/min.

Urinalysis showed leukocyturia, as indicated by both dipsticks analysis (+) and flow cytometry (65 m/l). Urine sediment examination identified glomerular red blood cells (43 m/l) and calcium oxalate monohydrate crystals. Further laboratory assessments showed elevated serum creatinine levels at 6.61 mg/dl (584 μmol/l), whereas venous blood pH was maintained at 7.42. A 24-hour urine sample demonstrated a slight rise in oxalate excretion at 426 μmol/24 h (normal range is 40–340 μmol/24 h) with normal calcium levels. Renal ultrasound imaging depicted normal kidneys with slight corticomedullary dedifferentiation, absence of pyelocaliceal dilation, and normal vascular flow. Hepatitis B and C viruses, along with HIV, were excluded. Autoimmune markers including PLA2R antibodies, antiglomerular basement membrane antibodies, antinuclear antibody, antineutrophil cytoplasmic antibody, and rheumatoid factor were negative. Complement serum levels (C3, C4, and CH50) were within normal range, and serum immunofixation did not reveal a monoclonal gammopathy.

**Figure 1:**
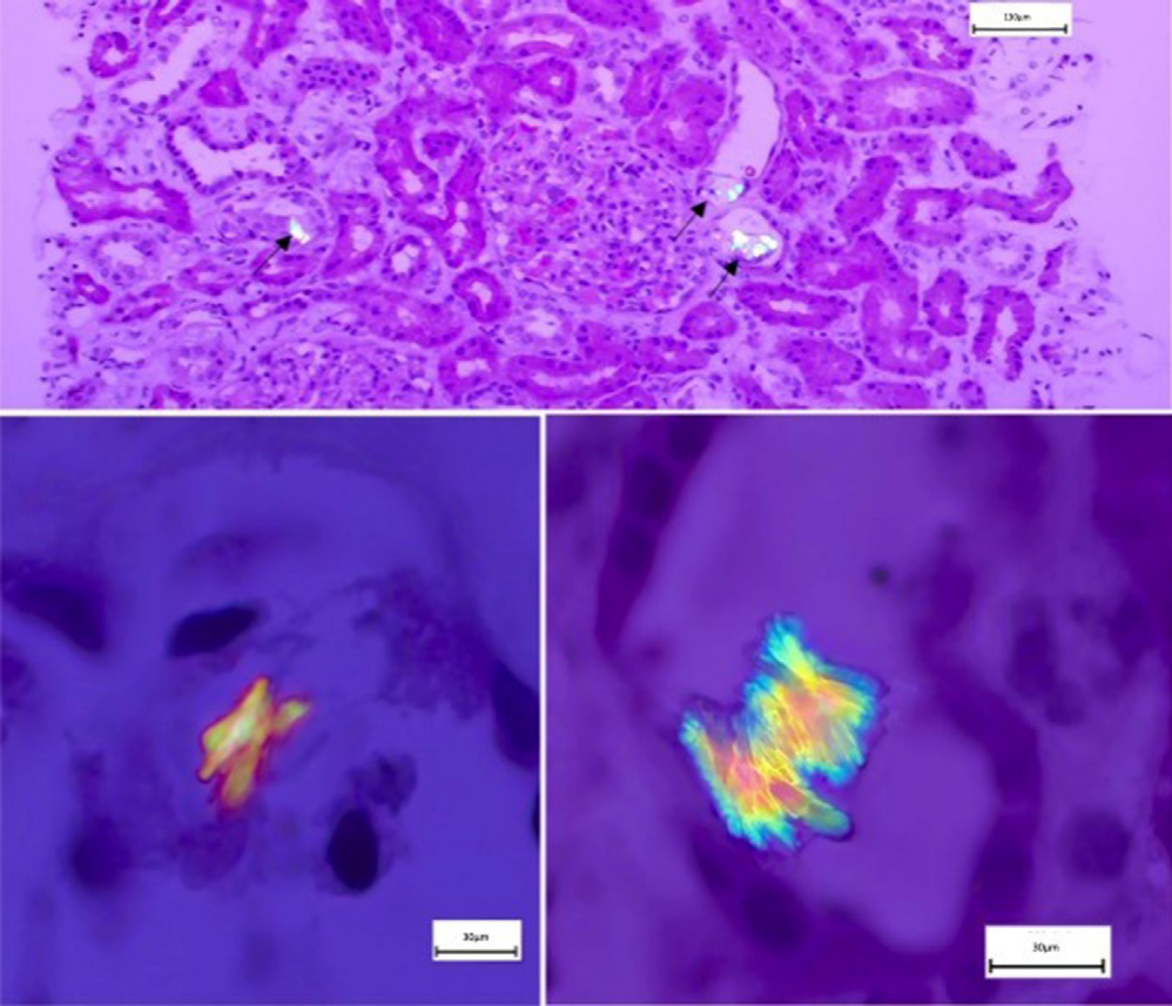
Histopathological examination using polarized microscopy. Kidney biopsy examined through polarized microscopy demonstrating birefringent crystals. The distinct “picket fence” morphology under polarized light suggests that these crystals may originate from the ethylene glycol metabolic pathway, consistent with glyoxylic acid intoxication. Further studies, however, are necessary to confirm whether this distinctive morphology, typically observed in urine analyses, is also present in kidney biopsies. Bar = 130 µm on top and 30 µm at the bottom.

Renal biopsy histology revealed numerous microcrystalline deposits of calcium oxalate (Figure 1). Furthermore, tubulointerstitial alterations composed of mild diffuse interstitial edema without inflammatory infiltrate were noted. Immunofluorescence and electron microscopy of the glomeruli showed no immune deposits and a normal glomerular basal membrane.

The patient received conservative management and was discharged after 4 days with a decreasing serum creatinine, 2.69 mg/dl (238 μmol/l). At 3-month follow-up, the patient’s serum creatinine further improved to 0.87 mg/dl (77 μmol/l) and a 24-hour urine sample indicated low oxalate excretion of 89 μmol/24 h and the absence of glomerular hematuria. Perindopril 10 mg daily was initiated for sustained hypertension.

On further questioning, the patient reported that she underwent hair straightening in Brazil before her return to Switzerland, and that symptom occurred subsequently.

## Discussion

Glycolic acid derivatives, such as glyoxylic acid, are used in keratin hair treatments as an alternative to formaldehyde to restructure and straighten hair by interacting with keratin fibers.^4^ This method is claimed to be superior to formaldehyde, known for its irritant properties and potential carcinogenicity.^5^ A recent study has shown that enhancing these treatments with additional glycolic acid can increase the absorption of glyoxylic acid, potentially improving their efficacy.^4^

After systemic absorption, glycolic acid is oxidized to glyoxylic acid by glycolate oxidase within hepatocytes. Alternatively, glyoxylic acid can be absorbed directly. Subsequently, glyoxylic acid, likely in the form of glyoxylate at physiological pH, can be further metabolized into oxalic acid by lactate dehydrogenase in the cytoplasm. At physiological pH of 7.4, oxalic acid dissociates to form oxalate ions, which can precipitate as calcium oxalate crystals within renal and other tissues.^6^, ^7^, ^8^ This metabolic pathway is analogous to that observed in ethylene glycol poisoning, where glyoxylic acid is an intermediary metabolite. Excessive vitamin C can similarly lead to oxalic acid production through another metabolic pathway. In Supplementary Figure S1, we summarize these biochemical processes. The calcium oxalate crystals identified in such instances are predominantly monohydrated, shuttle-shaped (Figure 1).

In this case study, we report a severe acute renal injury shortly after the use of hair straightening procedure in Brazil, potentially containing glyoxylic acid. Clinical presentation with nausea, vomiting, and abdominal pain suggested an intoxication. Viral, immunological, and pharmacological factors were excluded. Ultrasound imaging effectively ruled out postrenal etiology. Histopathological analysis revealed oxalate nephropathy. Moreover, the 24-hour urinary metabolic profile did not provide substantial evidence to suggest a dietary origin or sustained hyperoxaluria.

Our case closely aligns with the 27 cases previously reported in the scientific literature, as summarized in Supplementary Table S1. Histopathological and urine sediment findings support the hypothesis that AKI was induced by nephropathy due to oxalate deposits. The distinctive picket-like morphology and important number of these deposits indicate an etiology consistent with an intoxication pathway, in contrast to the dumbbell or oval-shaped calcium oxalate crystals typically associated with dietary causes.^9^ Nephrologists should, therefore, consider recent hair straightening treatments as a potential cause of severe AKI, especially when accompanied by scalp irritation.

In regions such as Brazil, Argentina, and North Africa, the use of glyoxylic acid-based products for hair straightening is increasingly popular, explaining why such cases are still rare in Europe and the United States. However, the introduction of cosmetic products containing these compounds could lead to significant health concerns. Given the established link between glyoxylic acid and oxalate nephropathy with severe AKI, avoidance of these substances is recommended.

To conclude, glyoxylic acid and its derivatives, widely considered safe, are extensively utilized in the cosmetic industry. These recent findings, however, suggest that its application, particularly in hair straightening procedures and potentially other cosmetic forms, could pose significant health risks and warrant cautious use. The occurrence of AKI in this patient, in the absence of other clear etiologies, underscores the need for health care providers to consider exposure to products containing glyoxylic acid, particularly when scalp irritation is present. Further research investigating the dermal absorption of glyoxylic acid is essential to assess the safety of these cosmetic applications.

## Disclosure

L.B. is supported by a grant from the 10.13039/501100001711Swiss National Science Foundation (PZ00P3_208670/1). A.H. is the recipient of a grant from the 10.13039/501100001711Swiss National Science Foundation (323530_221874).

## Patient Consent

The authors declare that they have obtained consent from the patient discussed in this report.

## Supplementary Material

Supplementary File (PDF) **Figure S1.** Metabolic pathway that can lead to oxalate deposits. **Table S1.** Clinical symptoms and signs.

## Footnotes

Supplementary File (PDF)
**Figure S1.** Metabolic pathway that can lead to oxalate deposits. **Table S1.** Clinical symptoms and signs.
