# Supplementary material for: Acute Kidney Injury and Hair-Straightening Products
Source: Kidney Int Rep. 2024 Jun 10;9(8):2571–3. doi: 10.1016/j.ekir.2024.06.010 (PMC11328560; doi:10.1016/j.ekir.2024.06.010)
Supplement: Supplementary File (PDF) — Figure S1. Metabolic pathway that can lead to oxalate deposits. Table S1. Clinical symptoms and signs. [file mmc1.pdf]

Table S1: Clinical symptoms and signs
(based on the 28 cases reported in scientific literature)

Nausea and/or vomiting	27 (96%)
Abdominal pain	12 (79%)
Flank pain	11 (43%)
Scalp rash	10 (36%)
Fever	2 (7%)
Headache	1 (4%)

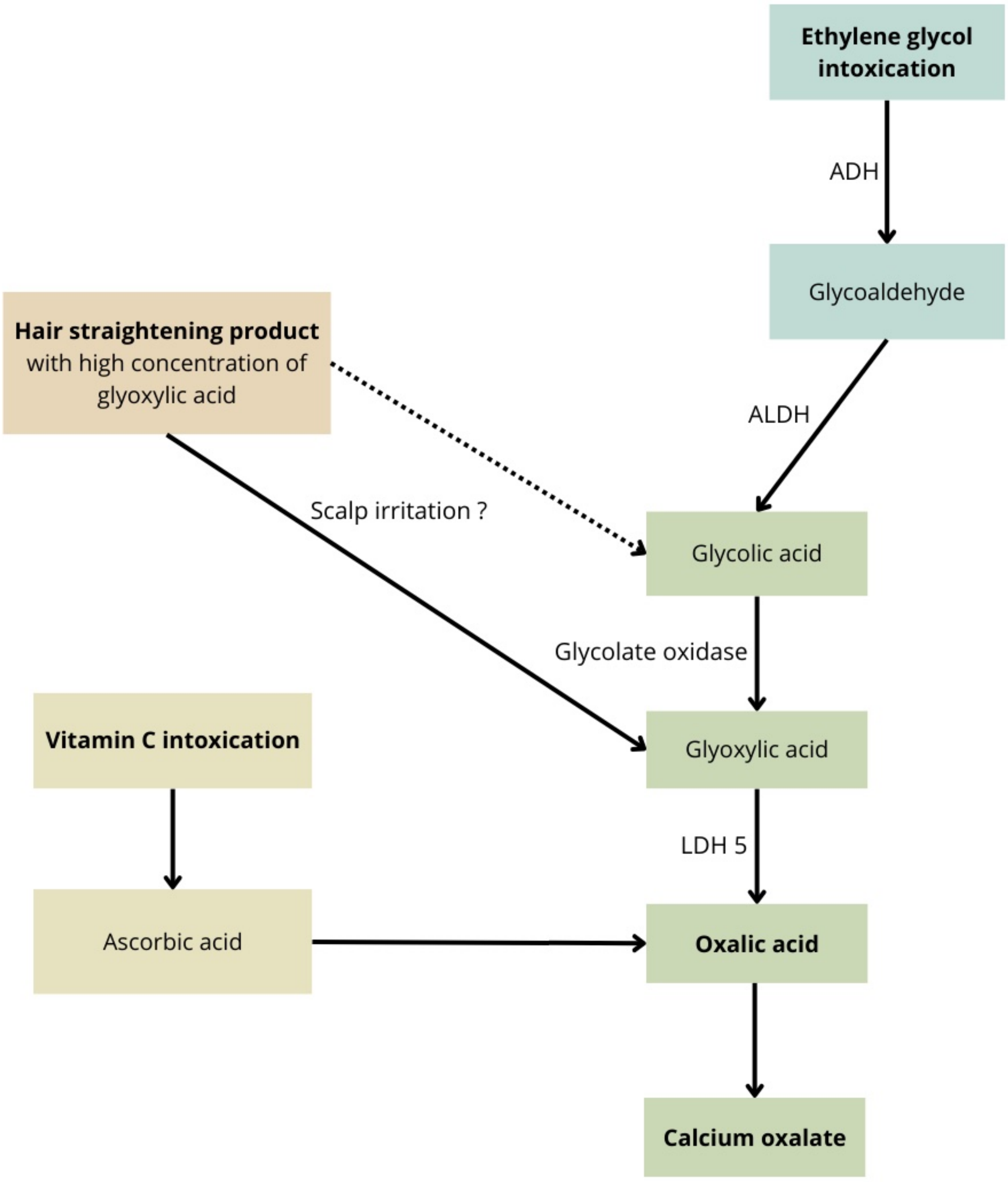

Figure S1: Metabolic pathway that can lead to oxalate deposits.

Calcium oxalate formation through three different pathways: ethylene glycol intoxication (blue), hair straightening products (orange), vitamin C intoxication (yellow). Solid arrows represent enzymatic reactions leading to product formation. Enzymes facilitating each step are denoted in plain text adjacent to the corresponding arrows: alcohol dehydrogenase (ADH), aldehyde dehydrogenase (ALDH), glycolate oxidase and lactate dehydrogenase (LDH 5).
